# 10-cm mature cystic teratoma of the bladder in 48-year-old female misdiagnosed as bladder calculus: Management with robotic partial cystectomy

**DOI:** 10.1016/j.eucr.2023.102320

**Published:** 2023-01-03

**Authors:** Benjamin J. Behers, Spencer B. Kortum, Isabella G. Bermingham, Karim Ghazli, Robert I. Carey

**Affiliations:** a Florida State University College of Medicine, Tallahassee, FL, USA; b Jellison Cancer Institute, Sarasota Memorial Hospital, Sarasota, FL, USA

**Keywords:** Mature cystic teratoma, Robotic partial cystectomy

## Abstract

Cystic teratomas are a common ovarian neoplasm that are rarely found in other locations of the body, namely the sacrococcygeal region and anterior mediastinum. Localization to the urinary bladder is exceedingly rare, with only a few cases documented in the literature. Cystic teratomas are usually asymptomatic and found incidentally, but localization to the bladder can present as irritative lower urinary tract symptoms and/or mimic urinary tract calculi. We report the rare case of a mature cystic teratoma of the urinary bladder, presenting as foul-smelling urine with recurrent urinary tract infections and microhematuria, that was originally misdiagnosed as a bladder calculus.

## Abbreviations

MCT: Mature Cystic Teratoma
UTIs: Urinary Tract Infections
ER: Emergency Room
CT: Computed Tomography

## 1 Introduction

Cystic teratomas are the most common ovarian neoplasm, with an estimated incidence of 1.2–14.2 cases per 100,000.^1^ While common in the ovary and testis, they are rarely found in other locations, including the sacrococcygeal region and the anterior mediastinum.^1^ Mature cystic teratomas (MCTs) are characterized by well-differentiated derivatives of germ cell layers, often developing as hair, muscle, teeth, or bone.^1^ Here, we report the rare case of a MCT of the urinary bladder, presenting as foul-smelling urine with recurrent urinary tract infections (UTIs) and microhematuria, originally misdiagnosed as a bladder calculus.

## 2 Case presentation

A 48-year-old Gravida 4, Para 4 Hispanic female presented to our Urology clinic for persistent foul-smelling urine. She denied symptoms of dysuria or fever. Past medical history was notable for recurrent UTIs and a 6-cm bladder calculus discovered during an Emergency Room (ER) visit for a complicated UTI three years prior. This was treated with cystoscopy laser lithotripsy by an outside urologist at that time. Of note, her urine grew extended-spectrum beta-lactamase (ESBL) *Escherichia coli* at this time. Her obstetric history is notable for three normal standard vaginal births and one Cesarean section with a bilateral tubal ligation, but she retained her ovaries. She has no family history of gynecologic or obstetric conditions.

**Fig. 1:**
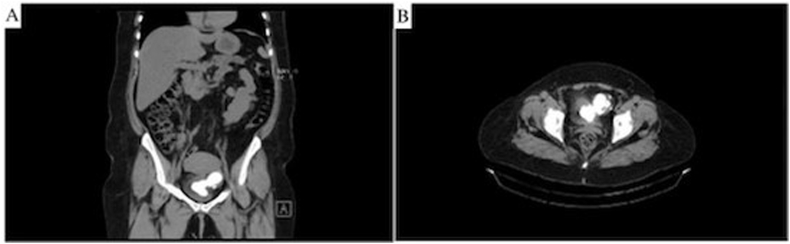
A) Coronal and B) Axial CT scans showing the bi-lobed 10-cm calculus in the bladder and its diverticulum.

Vital signs were within normal limits. Urinalysis was notable for large leukocyte esterase and small blood. Diagnostic cystoscopy appreciated a 10-cm calculus, or calcified mass, arising from the left wall of the bladder and extending deeply into the lumen of the bladder. An empty right-sided diverticulum was also observed. Computed tomography (CT) imaging showed a bi-lobed calcification radiographically interpreted as a 5-cm calculus in a left-sided bladder diverticulum with a 5-cm extension into the bladder (Fig. 1).

A plan was developed for her to undergo robotic-assisted laparoscopic partial cystectomy. This decision was made with the understanding that the lesion could be removed if it were either a solid mass or a stone in a diverticulum. Transperitoneal robotic surgery provided direct access to the bladder and adnexa without having to move any other structures. Supraumbilical camera port placement revealed the lesion for immediate inspection, and it was easy to triangulate the robotic arms for excision of the mass and repair of the bladder. First, the diverticulum and its calculus were completely excised from the urinary bladder. Intraoperative inspection of the presumed bladder calculus revealed that the bladder lesion did not look typical for a calculus; instead, its appearance favored an atypical solid tumor. The tumor was completely excised from the urinary bladder and retrieved (Video).

Supplementary video related to this article can be found at https://doi.org/10.1016/j.eucr.2023.102320

The following is the supplementary data related to this article:

Video Intra-operative video showing removal of the diverticulum with its calculus and the remaining calculus in the bladder. Video

**Fig. 2:**
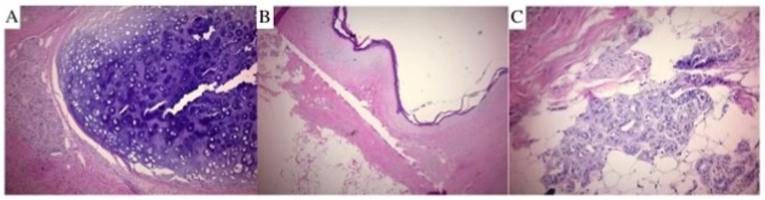
Histology pictures of the specimen on Hematoxylin and Eosin stain showing: A) Cartilage, B) Skin, and C) Apocrine Glands.

Both the diverticulum with calculus and additional calculus retrieved from the bladder were sent to pathology for analysis. Gross examination revealed two tan, irregular, hard calculi (4.2 × 3.5 × 3.2 cm and 5.5 × 4.9 × 4.2 cm). Histological examination found them to be a mature cystic teratoma with associated calcifications and clear margins. Cartilage, skin, and apocrine glands were observed throughout the lesion (Fig. 2). No atypia or malignancy was noted. Urine obtained from the bladder intra-operatively grew *Enterococcus faecalis.*

She tolerated the procedure well and was discharged the following day. At four-month follow-up, diagnostic cystoscopy noted a small residual bladder scar in the left wall of the bladder. Transurethral resection of the scar demonstrated areas of granulation tissue and calcifications, without recurrence of the teratoma and no malignancy. Her urinalysis was also negative; and she reported no recurrence of UTI symptoms and resolution of her foul-smelling urine.

## 3 Discussion

MCTs are the most common ovarian neoplasm; however, localization to the urinary bladder is exceedingly rare with few cases reported in the literature. This makes accurate diagnosis difficult. The clinical presentation, localization, and notable calcification appreciated on imaging studies create a pitfall in which a bladder calculus may appear a reasonable diagnosis. However, as seen in our case, the discovery of a different pathology only occurred intra-operatively. Surgical removal with diverticulectomy successfully removed the tumor, with histology determining it to be an MCT. Resolution of the patient's foul-smelling urine was achieved and there has been no subsequent UTIs in the six months following the procedure.

The first case of a mature cystic teratoma of the urinary bladder was published in 1981.^2^ This was in the form of a retrospective review of pediatric germ cell tumors over a 25-year period, only documenting location and type of tumor, but not offering a thorough presentation of the clinical course. Studies suggest that up to 65% of MCTs present asymptomatically and are discovered incidentally on imaging studies, although MCTs of the bladder may cause irritative lower urinary tract symptoms or urinary retention.^1^^,^^3^ Cases have also been reported describing MCTs of the bladder mimicking urinary tract calculi.^3^^,^^4^ However, to our knowledge, this is the first report suggesting a possible association between recurrent culture-proven UTIs and bladder teratomas. This is an important consideration for antibiotic stewardship and management of recurrent UTIs.

MCTs have an age-related incidence, most frequently occurring in female patients between 20 and 40 years of age with a median age of 30.^1^^,^^3^ They are usually benign and have a growth rate of 1.8 mm/year.^1^ Given the 10-cm size of the MCT reported here and the later age of presentation at 48-years-old, it is likely to have been there for years. It remains unclear whether this represented a primary bladder teratoma or a skip lesion from her ovary.

Treatment of MCTs involves complete removal of the tumor, with recurrence uncommon when performed successfully.^5^ Although malignant transformation is rare, further treatment is required in these situations, typically either chemotherapy or radiation.^5^ Robotic-assisted surgical removal, as was performed here, appears to be an effective treatment modality for MCTs of the urinary bladder.

## 4 Conclusions

Our case highlights the rare phenomenon of a mature cystic teratoma of the urinary bladder. The presentation mimics that of bladder calculi, making definitive preoperative diagnosis challenging. Mature cystic teratoma should be considered on the differential of perceived bladder calculi, especially in the setting of recurrent UTIs and prior unsuccessful lithotripsy. Treatment involves complete surgical removal of the MCT, preventing recurrence and alleviating the urinary symptoms.

## Color

We kindly ask that our media be printed in color.

## Consent

Informed, written consent was obtained from the patient for publication of this case.

## Funding

This research did not receive any specific grant from funding agencies in the public, commercial, or not-for-profit sectors.

## Declaration of competing interest

There are no conflicts of interest. The author(s) received no financial support for the research, authorship, and/or publication of this article.

## References

[1] Ahmed A., Lotfollahzadeh S. (2022).

[2] Marsden H.B., Birch J.M., Swindell R. (1981). Germ cell tumours of childhood: a review of 137 cases. J Clin Pathol.

[3] Omar M., El-Gharabawy M., Samir A., El Sherif E., Monga M. (2017). Mature cystitic teratoma of the bladder masquerading as a distal ureteral stone. Urol Case Rep.

[4] Okeke L.I., Ogun G.O., Etukakpan B.R., Iyama A., Adeoye A.O., Duduyemi B.M. (2007). Dermoid cyst of the urinary bladder as a differential diagnosis of bladder calculus: a case report. J Med Case Rep.

[5] Prihadi J.C., Kusumajaya C. (2018). Mature teratoma of the bladder in adolescence: a case report and literature review. Res Rep Urol.

